# Key Subordinate Executive Governance, CEO Overconfidence, and Accounting Conservatism: From the Perspective of Sustainable Development

**DOI:** 10.3389/fpsyg.2021.799221

**Published:** 2022-02-02

**Authors:** Fan Wu, Xuewen Kuang

**Affiliations:** School of Economics and Management, Nanchang University, Nanchang, China

**Keywords:** internal governance, TMT, overconfidence, sustainable development, accounting conservatism

## Abstract

Key subordinate executives play the role of connecting superiors and subordinates within the top management team (TMT). Based on the heterogeneity of TMT preference, this article takes the data of Chinese A-share listed companies from 2010 to 2019 as a sample to examine whether key subordinate executive governance can affect the short-sighted behavior of CEOs. The empirical result shows that there is a positive relationship between key subordinate executive governance and accounting conservatism, and CEO overconfidence can positively moderate the relationship. The study also shows that there is a significant positive relationship between key subordinate executive governance and accounting conservatism in private enterprises and enterprises with high market competition, that is, the key subordinate executives of these two types of enterprises can better enhance the conservatism under the stimulation of CEO overconfidence. This study contributes to the literature by examining how key subordinate executives affect accounting conservatism and link the prudential attitude of key subordinate executives with the behavioral tendency of CEO overconfidence, which has managerial implications for improving the power balance mechanism of TMT and strengthening the human resource incentive of key subordinate executives.

## Introduction

The agency theory states that the operator has different interest appeal and opportunistic behavior tendencies from the owner (Fama and Jensen, 1983). The core of corporate governance aims to solve the agency problem of such interest heterogeneity and form a supervision and restraint mechanism based on investors' interests (Berle and Means, 1932; Armstrong et al., 2010). However, existing research on corporate governance tends to regard the top management team (TMT) as a homogenous subject. In other words, the individual characteristics of the CEO represent the overall characteristics of the TMT to carry out research. The upper echelon theory argues that there are differences in age, tenure, and other demographic characteristics in the TMT (Hambrick and Mason, 1984), which lead to different value orientations among team members. The theory of team production points out that what a team provides is the product of the whole team, not the marginal product of a single member (Alchian and Demsetz, 1972). The heterogeneity of TMT will affect the decision-making process and results (Nielsen and Nielsen, 2013). Under the joint influence of previous theory, TMT is a collective organization, which not only has interest competition but also needs mutual assistance. Meanwhile, key subordinate executives, as the direct deputies of the CEO, are expected to become the CEO's successor. As a result, there are different career development opportunities between the candidate and incumbent, which lead to varying preferences for company value. The CEO, especially the outgoing CEO, pays more attention to the short-term performance of the company, while key subordinate executives, especially young and promising key subordinate executives, pay more attention to the long-term value of the company (Acharya et al., 2011). Therefore, based on the promising career prospects and ability to restrain team decision-making, key subordinate executive governance is a governance mechanism that check and balance the CEO's short-sighted behavior.

Accounting conservatism is not only an important requirement for the quality of accounting information, but also a key tool for the company's sustainable development. By not overestimating income or underestimating loss, it cautiously confirms an accounting element, alleviates the damage caused by information asymmetry to the investor (Watts, 2003), and maintains the sustainability of performance to support corporate value. The CEO is the direct leader of the company's operation, and the view of hierarchical authority under the Confucian tradition easily gives rise to the overconfidence of the CEO (Jiang et al., 2009), while the psychological characteristics of the CEO often determine the strategic decisions of the company (Chen et al., 2019). Malmendier and Tate (2008) pointed out that an overconfident manager is optimistic about corporate performance. An overconfident CEO, in turn, understates losses and overstates earnings, undermining accounting conservatism. Therefore, this kind of self-serving psychological behavior of the CEO leads the company to pursue short-term performance indicators, which violates the requirements of accounting conservatism and damages the long-term stable development of the company. For key subordinate executives who pay more attention to the long-term value of the company, it is bound to trigger the counterbalance to CEO overconfidence, thus forming the governance mechanism of key subordinate executives to the CEO.

This article has the following research contributions. First, this article improves the measurement of key subordinate executive governance. In the previous literature, the subordinate executive governance was generally measured from the perspective of age and salary (Cheng et al., 2016), it is believed that longer career prospects and contribution ability to the company could reflect the subordinate executive governance. However, these studies fail to consider a more substantive perspective, that is, corporate promotion mechanism and executive restraint mechanism. Based on institutional promotion incentives and practical supervision ability, this article extends the measurement indicators of key subordinate executive governance. Second, this article refines the subject of key subordinate executives. The subordinate executives involved in the previous literature are generally defined as the top four non-CEO executives (Jain et al., 2016). However, these studies overlook the special status of vice president (VP) after CEO. In this article, the key subordinate executives are defined as VPs, which improve the accuracy of the governance mechanism of key subordinate executives. Third, this article expands the governance role of key subordinate executives. Previous literature studies show that subordinate executives have governance effects on stock liquidity, earning management, and corporate social responsibility (Chen and Zhou, 2016; Cheng et al., 2016; Jain et al., 2016). However, it has not been examined how the subordinate executive governance affects accounting conservatism. This article links the cautious mentality of subordinate executives and the overconfident behavior tendency of the CEO to extend the research on the governance role of subordinate executives.

The remainder of this study is organized as follows. Section Literature Review and Hypotheses reviews the related literature and develops hypotheses. Section Research Design introduces sample selection, defines variables, and explains the empirical model. Section Empirical Results reports empirical examination results. Section Discussion offers research conclusions, summarizes contributions, and discusses implications and limitations.

## Literature Review and Hypotheses

### Key Subordinate Executive Governance

According to the Upper Echelons Theory proposed by Hambrick and Mason (1984), there are demographic differences among members of the TMT, such as age and gender, which also constitute the heterogeneity of the benefit orientation of team members. The heterogeneity determines the existence of cooperation and complementarity as well as restriction and supervision among the internal members of TMT. It requires both wise leadership from the CEO and a strong execution from the subordinate executive. At the same time, the subordinate executives, as potential CEO successors, have longer careers and are more focused on the long-term development of the company than the elder, outgoing CEO. As a result, the CEO is relatively focused on short-sighted interests, while key subordinate executives are relatively focused on far-sighted values. Fama (1980) pointed out that the effectiveness of the separation of ownership and operation right in modern companies lies in the combination of external and internal governance of the TMT, and there is a bidirectional supervision mechanism of top-down and bottom-up in the company. Acharya et al. (2011) proposed the concept of internal governance of the TMT, and regarded the TMT as an aggregate of different career visions and interest appeals. Jain et al. (2016) made it clear that this form of key subordinate executive governance is a bottom-up corporate governance mechanism. Cheng et al. (2016) further pointed out that subordinate executives would exert an influence on the CEO and urge them to restrain the tendency of short-sightedness and make decisions in line with long-term values, which construct the governance role for the CEO through supervision motivation and supervision ability.

The motivation of key subordinate executives to supervise the CEO is derived from the seniority order. As the leader of the TMT, the CEO is usually more senior and older than the subordinate executive. On the contrary, younger subordinates have a career advancement advantage over the CEO (Baker et al., 1988). Promotion of position is the incentive system under the concept of rank. Based on the psychological contract theory, employees have important expectations for job promotion (Morrison and Robinson, 1997). Possible job promotion in the future will greatly promote key subordinate executives to actively engage in their work and safeguard the interests of the company. Dencker (2009) pointed out that the psychological expectation that job promotion would bring higher returns drove subordinates to increase their work involvement. A good internal promotion mechanism in the company indicates that there are greater opportunities for promotion in the future, which potentially motivates subordinate executives to work hard and pay more attention to the long-term interests of the company. According to the tournament theory, the perceived probability of promotion is positively correlated with the effort invested to win the tournament (Kale et al., 2009). Key subordinate executives are the first echelon in the TMT to achieve generational change. They have a higher probability of being promoted to CEO and bring more incentive intensity than other members of the team. When key subordinate executives perceive higher promotion opportunities, the trophy incentive effect will promote them to improve risk-taking and corporate performance (Kato and Long, 2011; Kini and Williams, 2012). Therefore, key subordinate executives would pay more attention to the long-term interests of the company when there is a promising prospect within the company, i.e., when they have the opportunity to take over as CEO in the future.

Subordinate executives not only have the incentive to supervise the CEO but also have the actual ability to supervise the CEO (Acharya et al., 2011). Hambrick (1994) defined the behavioral integration of the TMT. The decision-making behavior within the team is not a one-way order but a two-way system of sharing and cooperation. On one hand, the CEO's current benefits depend on current cash flow, which is affected by the effort of the subordinate executive. As a result, if the CEO does' not consider the preferences of his subordinate executive when making decisions about how to run the company, his subordinate executive will probably not work hard. It is bound to reduce the company's current cash flow and CEO welfare, which Landier et al. (2009) call “executive constraints.” The essence of enterprise operation is team production (Alchian and Demsetz, 1972). TMT needs overall cooperation to achieve the output to the outside world. The realization of executive team creativity is also a process of collective effort and consideration of various opinions (Rong and Wang, 2021). As the direct deputy of the CEO, key subordinate executives play an important role in conveying and executing the orders made by the CEO. The CEO and key subordinate executives are the top two levels in the TMT, and their effective cooperation is essential for the smooth operation of the company. The CEO should not only rely on the information provided by his subordinates, but also consider 'subordinates' interests and preferences when making business decisions. If the CEO's decisions are short-sighted, self-serving, and damaging to the long-term value of the company, key subordinate executives focused on the long-term value of the company will not cooperate in the implementation, thus reducing future cash flow and CEO welfare. As a result, the executive team is built on the decisions of the CEO and the execution of key subordinate executives. This collaboration mechanism gives key subordinate executive selective execution space and regulatory execution strength, forming restrictive governance of CEO's self-interested behavior.

Acharya et al. (2011) pointed out that subordinate executives can make the CEO pay attention to far-sighted interests because they have a longer career horizon. Jain et al. (2016) emphasized the important influence of age on individual behavior and future aspirations, and researchers believed that the age difference between CEO and subordinates is key to the internal governance of TMT. Cheng et al. (2016) believed that the relative horizon adopted by Jain et al. (2016) is not accurate enough, and subordinates' own horizon is more important. Then, the supervisory motivation of key subordinate executives is captured by using their remaining career horizons. However, this literature's age-based measures are only part of the governance motivations. Aggarwal et al. (2017) found that when the CEO is an internal successor, key subordinate executives would pay more attention to the corporate long-term interests. Therefore, subordinates' career prospects depend not only on the horizon before retirement, but also on whether the next CEO is selected from within the subordinates. Indeed, this is also consistent with the implication of Acharya et al. (2011), and it is necessary to pay attention to the internal promotion mechanism to improve the governance of subordinate executives. A good internal promotion environment means more continuous institutional incentives, providing key subordinate executives with another part of the governance motivation.

Moreover, regarding the measurement of supervisory ability, Cheng et al. (2016) believed that compensation reflected their structural power within the enterprise, and then adopted the compensation ratio of subordinate executives to CEO to capture supervisory ability. Chen and Zhou (2016) likewise adopted relative salary. Aggarwal et al. (2017) adopted the number of titles to represent the relative contribution of TMT members. However, Acharya et al. (2011) also pointed out that supervision ability lies in subordinates' right to withdraw their contributions to the company. Therefore, how subordinates have supervision ability depends not only on the proportion of personal contributions made by subordinates, but also on the practical restraint ability imposed by subordinates. Antia et al. (2010) believed that tenure can reflect the company-specific experience and knowledge accumulated by executives. Subordinates are important to the daily operation of enterprises (Aggarwal et al., 2017), so that the experience behind subordinates' tenure can affect the practical implementation of CEO decisions. In addition, Finkelstein (1992) pointed out that board membership means the core member of the company, and is responsible for the final decision of the company's policies. As a result, subordinates who are also directors can influence the core content of the CEO's decisions. Subordinate executives can obtain the important supervisory ability to influence decisions based on their personal qualifications and core identities. Such substantive constraints mean a more effective check-and-balance mechanism, providing another part of the governance ability of key subordinate executives.

In addition, previous literature studies generally defined key subordinate executives as the top four non-CEO executives (Chen and Zhou, 2016; Cheng et al., 2016; Jain et al., 2016). However, VPs have better promotion opportunities and more direct supervision. In the study of Bognanno (2001), the VP is regarded as an important competitor for the CEO position. Lin et al. (2011) listed VP as the second tier of the senior executive after CEO in the research of Chinese listed companies. Subordinate executives have an important influence on company's decision-making (Hambrick and Mason, 1984). Further, the VP is the immediate subordinate of the CEO and reports to the CEO directly, thus having a more direct influence. Therefore, VPs and other subordinate executives are not homogenous subjects, and their key governance position within the TMT has not been paid attention to by existing research on TMT internal governance.

By reviewing and analyzing the previous literature research, the component variables of key subordinate executive governance include the factors of internal promotion environment and practical constraint ability, and the research subject of key subordinate executives is defined as VP, to carry out follow-up research.

### Accounting Conservatism

Basu (1997) defined accounting conservatism as the asymmetric recognition of gains and losses. Accounting conservatism is also the principle of prudence. On one hand, the recognition of income is required to be higher than expenses; on the other hand, assets and income should not be overestimated, while liabilities and expenses should not be underestimated (Basu, 1997). Accounting conservatism also promotes the loss of bad news to be included in earnings faster than expected, and the gain of good news to be included in earnings slower than expected (Guay and Verrecchia, 2018). Based on the agency theory, the CEO manipulates earnings to cope with performance pressure and seize a competitive position (Kasznik and McNichols, 2002; Durana et al., 2021), especially upward earnings management for inflated accounting earnings (Cheng et al., 2016). However, the essential characteristics of accounting conservatism can restrain CEO's short-sighted self-interest psychology. Considering this governance function of accounting conservatism (Ball et al., 2000) and as an important representation of corporate agency problems, many scholars have studied accounting conservatism from the perspective of internal governance. Lafond and Roychowdhury (2008) investigated the impact of management shareholding on accounting conservatism. With the decrease of management shareholding, which runs counter to the interests of shareholders, the serious agency problem increases the demand for accounting conservatism of companies. Cullinan et al. (2012) studied accounting conservatism from the perspective of ownership structure based on Chinese samples. The decline of accounting conservatism reflects the aggravation of agency problems of major shareholders. Sultana (2015) focuses on accounting conservatism from the perspective of the audit committee, which can curb the CEO's opportunistic behavior and restrain the CEO's tendency to exaggerate earnings.

Accounting conservatism includes conditional conservatism (CAC) and unconditional conservatism (UCAC). CAC, based on asymmetrical recognition criteria of gains and losses (Ball and Shivakumar, 2005), is a flexible adjustment made in response to changes in external information, so it is also known as *ex-post* conservatism. Beaver and Ryan (2005) pointed out that non-CAC is *ex-ante* conservatism, a conservatism policy that is decided during the initial confirmation of assets and liabilities, that is, before external news emerges. UCAC is an internal institutional setting independent of changes in the external environment. Qiang (2007) pointed out that the perspectiveness of UCAC enables companies to better deal with the arrival of bad news, identify bad news in advance to reduce litigation costs, promote the expensing of R&D expenditures to avoid future impairment, and achieve smooth and stable impairment ahead of bad news. UCAC is also an important part of accounting slack (Beaver and Ryan, 2005), which accelerates the recognition of expenses and delays the recognition of income, creating institutional redundancy space for the company's financial data. Because UCAC is based on the characteristics of the company's internal system, it is also more consistent with the ideological connotation of long-term institutional supervision over CEO by key subordinate executive governance, so this article focuses on UCAC.

Chen and Zhou (2016) pointed out that younger subordinate executives exert an influence on the CEO to restrain the short-sighted behavior of the CEO and promote the CEO to make more far-sighted decisions. In fact, the psychological characteristics of subordinate executives are consistent with the connotation of accounting conservatism. Improving accounting conservatism means timely identification of losses and reducing the uncertainty of future bad news for the company (Kim and Pevzner, 2010), so as to avoid the adverse consequences of future stock price collapse caused by the accumulation of hidden bad news (Kim and Zhang, 2016). Strengthening the accounting conservatism can enhance the certainty of the future development of the company, which is conducive to the sustainable growth of company value. In addition, Cullinan et al. (2012) found that internal power checks and balances could improve accounting conservatism. Therefore, the checks and balances exerted by subordinate executives are conducive to restraining the short-sighted behaviors of the CEO and further improving corporate accounting conservatism.

To seek a bright prospect of personal career, key subordinate executives pay more attention to the future development interests of the company and promote the improvement of corporate accounting conservatism by curbing the short-sighted self-interest behavior of the CEO. Hu et al. (2020) pointed out that managers' awareness of risk avoidance can enhance accounting conservatism. In other words, the higher the governance degree of key subordinate executives, the more supervisory motivation subordinate executives have, and the more likely they are to restrain CEO's short-term aggressive value through subordinate executives' long-term stable value, thus prompting the whole TMT to follow the concept of sustainable accounting conservatism. Meanwhile, key subordinate executives, who occupy a unique position in TMT, are responsible for directly executing the CEO's decision, so they have the constraint ability of decision execution, and can check and balance the CEO's short-sighted radical opportunistic behavior. Therefore, key subordinate executives not only have a longer career and a more prudent attitude toward accounting information confirmation, but also have the practical implementation constraints ability, thus restraining the CEO's short-sighted behavior of exaggerating earning. Depending on supervisory motivation and supervisory ability, key subordinate executive governance can accelerate the recognition of expenses and delay the recognition of revenue, thus improving corporate accounting conservatism. Based on the above literature review and theoretical analysis, this article puts forward the following research hypothesis:

*Hypothesis 1: Key subordinate executive governance is positively related to accounting conservatism*.

### The Moderating Role of CEO Overconfidence

Agent theory focuses on the self-interested behavior of the CEO and regards them as a completely rational economic person to carry out uniformly optimal behavior. In fact, bounded rationality is hard to avoid (Simon, 1955), and overconfidence is the most common cognitive bias of managers. In the hubris hypothesis proposed by Roll (1986), it is pointed out that managers' overconfidence will overestimate corporate earnings through the study of managers' overconfidence and acquisitions. As the leader of the enterprise operation, compared with ordinary people or subordinate executives, the CEO tends to be overconfident (Cooper et al., 1988; Nofsinger, 2005). Considering that Chinese enterprises will also be influenced by the hierarchical authority concept of Confucian culture, the over-recognition of self-ability brought by the CEO's high status will further strengthen the overconfidence psychology of the CEO (Jiang et al., 2009). Therefore, under the behavior tendency of CEO overconfidence, enterprises confirm in advance or overestimate earnings and delay confirmation or underestimate losses, which aggravates information asymmetry and damages investors' interests.

As an inherent psychological feature, CEO overconfidence is not easy to be accurately captured. Previous literature mainly measures CEO overconfidence based on stock options, and there are two measurement perspectives. From the perspective of delayed execution, the CEO is optimistic about the future prospects of the enterprise and delays the execution of fully exercisable stock options (Campbell et al., 2011; Reyes et al., 2020). From the perspective of over-holding, the CEO has confidence in their own management ability and holds stock options beyond the optimal level (Kim et al., 2016; Schumacher et al., 2020). There are also some literature studies that measure CEO overconfidence based on media reports, and evaluate CEO self-confidence from the description vocabulary of the CEO in mainstream media (Chen et al., 2015; Schumacher et al., 2020). However, based on China's institutional environment, the stock option incentive system is still underdeveloped, and the media's evaluation of CEO is relatively simple and scarce, which makes it difficult to form an effective measure of CEO overconfidence. The measurement method based on CEO's personal characteristics is relatively more stable and reliable. Therefore, this article uses gender, age, educational background, and CEO duality to measure CEO overconfidence (Chen and Chen, 2021).

Subordinate executives are willing to urge the CEO to act in a more far-sighted way (Acharya et al., 2011), thus promoting accounting conservatism in line with sustainable development. However, the CEO has a strong tendency of overconfident behavior (Nofsinger, 2005). Overconfident people have better-than-average psychological effects than others (Cormier et al., 2016), while overconfident managers tend to view the probability of enterprise success with excessive optimism, resulting in overestimating profits and underestimating losses (Heaton, 2002; Malmendier and Tate, 2008). In addition, to maintain personal welfare and seek career stability, the CEO will not timely disclose bad news, such as losses, but actively disclose good news, such as earnings (Kothari et al., 2009). This is contrary to the requirement of accounting conservatism, which emphasizes neither overestimating gains nor underestimating losses. Therefore, the behavior tendency of CEO overconfidence is detrimental to corporate accounting conservatism. In the face of this behavior tendency, key subordinate executives with longer careers need to strengthen the governance level to ensure corporate sustainable development.

CEO overconfidence is a latent cognitive bias, which is not easily identified by the outside world. The external supervision of the company fails to effectively deal with the problem of management overconfidence (Schrand and Zechman, 2012). As a team partner who works directly with the CEO, key subordinate executives are more likely to detect CEO overconfidence and short-sighted behavior tendency. Specifically, when the CEO gradually shows the behavior tendency of overconfidence, exaggerates the accounting income, and grabs the short-term self-interest, key subordinate executives will also strengthen governance and prudence to seek long-term development. The behavior tendency of CEO overconfidence positively stimulates the motivation of key subordinate executives to conduct governance, thus improving the corporate accounting conservatism level under the team rivalry. Therefore, this article proposes the following research hypothesis:

*Hypothesis 2: CEO overconfidence positively moderates the relationship between key subordinate executive governance and accounting conservatism. Other conditions being equal, the greater the CEO overconfidence, the stronger the positive relationship between key subordinate executive governance and accounting conservatism*.

## Research Design

### Sample Selection

In the context of Chinese institutional culture, the CEO tends to form the personal characteristics of overconfidence. At the same time, the fast-growing Chinese economy has promoted the rapid development of Chinese enterprises. The scale of enterprise has expanded, market competition has intensified, and key subordinate executives have become increasingly important to the CEO. Therefore, there are strong conflicts of interest among TMT members of Chinese listed companies, which provide an institutional environment for further research. Therefore, this article selects the data of Chinese A-share (Shanghai and Shenzhen Stock Exchanges) listed companies from 2010 to 2019 as the initial research sample, and the related data of listed companies are mainly from The CSMAR database. ST and ^*^ST companies were excluded, as were financial industry companies, insolvent companies, and industries with less than 10 companies. Additionally, data missing samples were excluded. To eliminate the effect of extreme values, all the continuous variables at the 1st and 99th percentiles were trimmed.

### Variable Definitions and Regression Model

#### Independent Variable: Key Subordinate Executive Governance

Because the deputy general manager has a unique career development status different from other subordinate executives, this article defines key subordinate executives as the direct deputy of the CEO, namely the VP. And, the governance supervision of key subordinate executives is subdivided into two aspects, namely supervision motivation and supervision ability, so that the key subordinate governance mechanism can be better constructed comprehensively, and specific indicators are defined in Table 1.

**Table 1:** Definition of variables.

**Variable type**	**Variable name**	**Quantitative standard**	
Dependent variable	UCAC	Non-operating accruals of the current period divided by total assets of the previous period, multiplied by (−1)	
Independent variable	CGI	Supervision	Internal selection tradition of CEO (more than half of the CEO are selected from within the company)
		motivation	Setting up the position of EVP
			Internal selection tradition of chairman (more than half of the chairmen are selected from within the company)
			Subordinates career length (remaining years before the subordinates reach the age of 60)
		Supervision ability	The number of subordinate executives concurrently serving as directors
			The number of subordinate executives' positions
			The tenure time of subordinate executives
			salary ratio between subordinates and CEO
Moderating variable	overconfid	CEO's personal characteristics (gender, age, educational level and CEO duality)	
Control variables	size	Log value of corporate total assets	
	lev	Total liabilities scaled by total assets	
	EPS	Ratio of net profit to its number of shares	
	TBQ	Ratio of a company's market value to its total assets	
	PPE	Fixed assets scaled by total assets	
	balance	Ratio of the shares of the second to tenth largest shareholders to the first largest shareholder	
	indep	Proportion of independent directors on the board	
	HHI	Herfindahl-Hirschman index of product market competition	
	insthold	Shareholding ratio of institutional investors	

##### Supervision Motivation

In the aforementioned studies, supervisory motivation is generally measured by the remaining horizon (Cheng et al., 2016). Based on this, this article also considers the internal selection tradition of the CEO, the internal selection tradition of the chairman, and the position setting of executive vice president (EVP) as measurement standards. In addition, as a successor to the CEO position, the EVP often plays a transitional role between the deputy general manager (VP) and the CEO (Xu, 2012), and the position setting of the EVP can effectively encourage the VP to play the role of bottom-up supervision.

##### Supervision Ability

Supervision ability is generally measured by the ratio of subordinates' compensation to CEO's compensation (Cheng et al., 2016), the contribution of number of titles is considered (Aggarwal et al., 2017). On one hand, high seniority represents the rich company-specific experience owned by subordinates (Antia et al., 2010), on the other hand, the board of directors empowers directors to make decisions (Finkelstein, 1992). Therefore, key subordinate executives have the practical constraints to influence decision-making based on their personal qualifications and core identity. This article also includes the tenure time of key subordinate executives, the number of key subordinate executives concurrently serving as directors.

After that, the continuous variable indicators of key subordinate executive governance are normalized by min-max, which is convenient for subsequent weighting processing. (1) Under the simple weighting method, all indexes are directly added according to equal weights, and then obtained according to their arithmetic average; (2) Under the principal component analysis, each principal component is weighted according to its own contribution rate of variance, and then added to get it; According to the abovementioned assignment method, the comprehensive indexes CGI (*CGI_comp, CGI_pca*) of key subordinate governance are obtained.

#### Dependent Variable: UCAC

According to the research of related scholars (Givoly and Hayn, 2000; Zhang and Wang, 2013), in this article, the UCAC (*UCAC*_*i, t*_) under the vision of cumulative accruals is defined as follows:


(1) $$\begin{array}{l} UCAC_{i,t}=-NOPACC_{i,t}/TA_{i,t-1} \end{array}$$

(2) $$\begin{array}{l} NOPACC_{i,t}=Total\;Accruals_{i,t}\\ \;-Operating\;Accruals_{i,t} \end{array}$$

(3) $$\begin{array}{l} Total\;Accruals_{i,t}+Net\;Income_{i,t}+Depreciation_{i,t}\\ \;-{Cash\;Flow\;from\;Operations}_{i,t} \end{array}$$

(4) $$\begin{array}{l} Operating\;accruals_{i,t}=\Delta Accounts\;Receivable+\Delta Inventories\\ \;+\Delta Prepaid\;Expenses-\Delta Accounts\;Payable\\ \;-\Delta Taxes\;Payable \end{array}$$

In Equation (1), *UCAC*_*i, t*_ represents the UCAC in the current period, *NOPACC*_*i, t*_ represents the non-operating accruals in the current period, *TA*_*i, t*−1_ represents total assets in the prior period. Equations (2)–(4) define nonoperating accruals, total accruals, and operating accruals, respectively.

#### Moderating Variable: CEO Overconfidence

Key subordinate governance is a kind of continuous and stable institutional supervision, and the VP and CEO are interdependent and the balanced forces in the TMT. Therefore, based on theoretical analysis and institutional environment, this article adopts the personal characteristics of CEO to measure (Chen and Chen, 2021), namely four indicators including gender, age, educational level, and CEO duality, and takes their arithmetic average as the comprehensive score of overconfidence, the details are as follows: (1) Gender, men are more risky than women, variable equal to 1 if the CEO is male, otherwise it is 0; (2) Age, young people are more radical than old people, age is normalized by min-max, and the variable value is between 0 and 1; (3) Educational level, the higher the education level, the more confident they are, variable equal to 1 if the education level of CEO is a bachelor degree or above, otherwise it is 0; (4) CEO duality, if the CEO is also the chairman, show more confidence in their abilities, variable equal to 1 if the CEO concurrently serves as chairman, otherwise it is 0.

#### Control Variables

According to relevant research on corporate governance, in this article, company size, Tobin's *q* value, earnings per share, asset-liability ratio, tangible capital intensity, institutional investor shareholding, equity balance, the proportion of independent directors, market competition are selected as control variables, the specific definitions are shown in Table 1. In addition, the article also controls the industry fixed effect and year fixed effect, and further conducts clustering processing for individual and year.

### Regression Model

To test Hypotheses 1 and 2, this article constructed regression models (5)–(6) for empirical test:


(5) $$\begin{array}{l} UCAC_{i,t}=\beta_{0}+\beta_{1}CGI_{i,t}+\beta_{2}size_{i,t}+\beta_{3}TBQ_{i,t}+\beta_{4}EPS_{i,t}\\ \;+\beta_{5}lev_{i,t}+\beta_{6}PPE_{\text{i},\text{t}}+\beta_{7}insthold_{i,t}+\beta_{8}balance_{i,t}\\ \;+\beta_{9}indep_{i,t}+\beta_{10}HHI_{i,t}+\Sigma Ind+\Sigma Year+\varepsilon_{i,t} \end{array}$$

(6) $$\begin{array}{l} UCAC_{i,t}=\beta_{0}+\beta_{1}CGI_{i,t}*overconfid_{i,t}+\beta_{2}size_{i,t}*overconfid_{i,t}\\ \;+\beta_{3}TBQ_{i,t}*overconfid_{i,t}+\beta_{4}EPS_{i,t}*overconfid_{i,t}\\ \;+\beta_{5}lev_{i,t}*overconfid_{i,t}+\beta_{6}PPE_{\text{i},\text{t}}*overconfid_{i,t}\\ \;+\beta_{7}insthold_{i,t}*overconfid_{i,t}+\beta_{8}balance_{i,t}*overconfid_{i,t}\\ \;+\beta_{9}indep_{i,t}*overconfid_{i,t}+\beta_{10}HHI_{i,t}*overconfid_{i,t}\\ \;+\Sigma Ind+\Sigma Year+\varepsilon_{i,t\;} \end{array}$$

In model (5), *UCAC*_*i, t*_ represents the UCAC, *CGI*_*i, t*_ represents the governance level of key subordinates, and *CGI_comp* is the main variable in principal regression. Moreover, to enhance the robustness of results, *CGI_comp* is replaced by *CGI_pca* and added into regression. Model (6) based on model (5), focuses on the moderating mechanism of CEO overconfidence (*overconfid*), and analyzes the moderating effect whether the company has high CEO overconfidence.

## Empirical Results

### Descriptive Analysis

Descriptive statistics of relevant variables of the regression model were conducted in this article, and the statistical results are shown in Table 2. In the sample companies, the median and mean values of key subordinate governance indices whether weighted by the simple weighting method (*CGI_comp*) or principal component analysis method (*CGI_pca*) are at the level of 0.5. It shows that in listed companies, the overall level of key subordinate governance tends to be medium, and the range of maximum and minimum values is relatively concentrated and stable. The *UCAC* of the sample companies was positive or negative, which was consistent with the actual situation of the companies.

**Table 2:** Descriptive statistics of main variables.

**Variable**	**Mean**	**p50**	**Min**	**Max**	**SD**
UCAC	0.002	0.002	−0.312	0.337	0.094
CGI_comp	0.451	0.461	0.251	0.593	0.077
CGI_pca	0.498	0.502	0.187	0.768	0.126
overconfid	0.665	0.644	0.294	0.938	0.153
State	0.310	0.000	0.000	1.000	0.463
HHI	0.070	0.017	0.008	0.704	0.120

### Correlation Analysis

Table 3 reports the correlation analysis among the main concern variables. Key subordinate governance is positively correlated with accounting conservatism, and CEO overconfidence is positively correlated with accounting conservatism and key subordinate governance. The analysis results are close to the previous assumptions. In addition, this article also conducted a variance inflation factor (VIF) test on key subordinate governance and control variables. The VIF value of the independent variables is lower than 10, and there is no collinearity problem among independent variables.

**Table 3:** Correlation analysis of main variables.

**Variable**	**UCAC**	**CGI_comp**	**CGI_pca**	**overconfid**	**state**	**HHI**
UCAC	1.000					
CGI_comp	0.048^***^	1.000				
CGI_pca	0.059^***^	0.882^***^	1.000			
overconfid	0.049^***^	0.097^***^	0.132^***^	1.000		
state	−0.084^***^	−0.236^***^	−0.196^***^	−0.127^***^	1.000	
HHI	0.014^**^	−0.055^***^	−0.024^***^	−0.019^**^	0.101^***^	1.000

***, **, and * refer to significance at 1%, 5%, and 10%, respectively.

### Hypothesis Testing

#### Regression Analysis of Key Subordinate Governance and Accounting Conservatism

This article adopts the simple weighting method (*CGI_comp*) and principal component analysis (*CGI_pca*) simultaneously, and the key subordinate governance indexes weighted by the two methods were used as the independent variables. Furthermore, the *UCAC* and *CAC* were used as the dependent variables. The regression results of key subordinate governance and unconditional robustness are shown in columns (1) and (2) of Table 4. The regression coefficients are 0.028 and 0.016, respectively, and the significance level reaches 5%. Meanwhile, the sign of the regression coefficients is positive. The regression results show that when controlling for other related variables, there is a positive correlation between the key subordinate governance and the unconditional accounting conservatism. This regression result also supports Hypothesis 1. In addition, a comparative study is also carried out between different accounting conservatism. This article studies the relationship between key subordinate governance and CAC. The C-Score model of Khan and Watts (2009) is adopted to measure CAC. The corresponding model (9) was set for a comparative regression test.


(7) $$\begin{array}{l} \frac{EPS_{i,t}}{P_{i,t-1}}=\;\beta_{0}+\beta_{1}D_{i,t}+\;\beta_{2}R_{i,t}+\beta_{3}D^{*}R_{i,t}+\;\varepsilon_{i,t} \end{array}$$

(8) $$\begin{array}{l} CAC\equiv \;\beta_{3}=\lambda_{0}+\lambda_{1}Size_{i,t}+\lambda_{2}BM_{i,t}+\lambda_{3}lev_{i,t}+\varepsilon_{i,t} \end{array}$$

(9) $$\begin{array}{l} CAC_{i,t}=\beta_{0}+\beta_{1}CGI_{i,t}+\;Controls+\Sigma Ind+\Sigma Year\\ \;+\;\varepsilon_{i,t} \end{array}$$

*EPS*_*i, t*_ represents earnings per share of the current year, *P*_*i, t*−1_ represents the closing price of the stock on 1st May of the reporting year; *R*_*i, t*_is the annual stock returns calculated from 1st May of the reporting year to 30th April of the current year. If *R*_*i, t*_ < 0, *D* is 1; otherwise, it is 0. *size*_*i, t*_ is measured by the natural logarithm of the total market value of the firm; *BM*_*i, t*_ is the company's current year's price-to-book ratio; *lev*_*i, t*_ is the company's debt to asset ratio for the current year; β_3_ is to measure the difference between how much earnings reflect bad news and how much earnings reflect good news, β_3_ was defined as CAC. Then CAC (*CAC*_*i, t*_) was used as the explained variable, and the regression results were shown in columns (3) and (4) of Table 4. It is not statistically significant, but the regression coefficient is negatively correlated, indicating that key subordinate governance may also inhibit CAC. Therefore, UCAC is used to analyze accounting conservatism in subsequent studies.

**Table 4:** Key subordinate executive governance and accounting conservatism.

**Variables**	**Dependent variable:UCAC**		**Dependent variable:CAC**	
CGI_comp	0.028^**^		−0.155	
	(3.06)		(−1.74)	
CGI_pca		0.016^**^		−0.110
		(2.42)		(−1.66)
State	−0.007^**^	−0.007^**^	0.024	0.024
	(−3.20)	(−3.21)	(1.31)	(1.31)
HHI	−0.013	−0.013	−0.008	−0.006
	(−0.86)	(−0.87)	(−0.04)	(−0.03)
Constant	−0.049	−0.043	5.315^***^	5.297^***^
	(−1.19)	(−1.03)	(3.54)	(3.51)
Controls	Yes	Yes	Yes	Yes
Year	Yes	Yes	Yes	Yes
Ind	Yes	Yes	Yes	Yes
*N*	17,742	17,742	13,991	13,991
Adj R^2^	0.181	0.181	0.174	0.174

***, **, and * refer to significance at 1%, 5%, and 10%, respectively.

#### The Analysis of a Moderating Effect of CEO Overconfidence

As previously analyzed, under the influence of bounded rationality, CEO will show the psychological behavior characteristics of overconfidence, and it is easier to recognize revenue in advance or overestimate earning, thus damaging corporate accounting conservatism. As the CEO shows the behavior tendency that damages the long-term value of the company, the key subordinate executives will also strengthen their supervision ability, and form the effect of counterbalancing the CEO's related behavior tendency within the TMT.

Based on the above measures of overconfidence, companies whose overall overconfidence score is higher than the quartile (75%) of the sample are divided into CEO overconfidence group. Companies less than the quartile of the sample (25%) were classified as non-CEO overconfidence group. The independent variable (*CGI_comp*) is the level of key subordinate governance, and the dependent variable is unconditional accounting conservatism. The moderating mechanism of CEO overconfidence under different property right characters and different industry competition scenarios is further investigated. The regression results are shown in Table 5. There is a significant positive correlation between key subordinate governance and unconditional accounting conservatism at the level of 1% in the overconfidence group in column (2), while there is no significant correlation in the non-overconfidence group in column (1).

**Table 5:** Moderating effect of CEO overconfidence.

**Variables**	**Dependent variable:UCAC**					
	**Non-Overconfidence**	**Overconfidence**	**Overconfidence**			
			**State-Owned**	**Private-Owned**	**High-HHI**	**Low-HHI**
CGI_comp	0.008	0.047^***^	0.033	0.049^***^	0.075^***^	0.031
	(0.28)	(4.59)	(1.75)	(4.05)	(5.60)	(0.72)
State	−0.006^*^	−0.007^**^			−0.006	−0.008
	(−2.16)	(−3.08)			(−0.89)	(−1.21)
HHI	−0.008	−0.026	−0.068	0.000		
	(−0.23)	(−1.27)	(−1.37)	(0.01)		
Constant	−0.033	−0.053	0.027	−0.083	0.011	−0.050
	(−0.97)	(−0.89)	(0.35)	(−1.35)	(0.09)	(−0.62)
Controls	Yes	Yes	Yes	Yes	Yes	Yes
Year	Yes	Yes	Yes	Yes	Yes	Yes
Ind	Yes	Yes	Yes	Yes	Yes	Yes
N	3553	6974	2144	4828	1651	1802
Adj R^2^	0.206	0.177	0.177	0.181	0.293	0.160

***, **, and * refer to significance at 1%, 5%, and 10%, respectively.

In China's bureaucratic structure, such as state-owned enterprises, seniority is respected and hierarchy is emphasized (Du et al., 2017), which makes it difficult to play the governance mechanism of key subordinate executives. Therefore, this article further subdivides the CEO overconfidence group into the state- and private-owned group, and the regression results are shown in columns (3) and (4) of Table 5. In columns (1) and (2), the state-owned enterprise variable (*state*) is negatively correlated with accounting conservatism. The regression results of column (4) private enterprise group and column (3) state-owned enterprise group show that the key subordinate executives of private enterprise are more likely to restrain the overconfidence of CEO, so as to positively strengthen the accounting conservatism of enterprises.

In addition, external industry competition can strengthen the level of corporate governance. On one hand, Kliestik et al. (2021) believe that the external environment of enterprises will affect the reliability of financial data. On the other hand, the study of Yang and Xu (2020) point out that industry competition and internal governance mechanisms have a synergistic regulatory effect on opportunistic behaviors of management. Schmidt (1997) believes that industry competition can strengthen and motivate managers' operating capacity. Chen et al. (2013) also point out that industry competition has enhanced the motivation of shareholders to supervise the opportunistic behaviors of management. Therefore, the strength of external industry competition plays an important role for key subordinate executives to supervise and restrain the CEO governance mechanism. In this article, the CEO overconfidence group is further divided into high-Herfindahl–Hrschman index (HHI) and low-HHI, respectively, which are higher than the upper quartile and less than the lower quartile. The regression results are shown in columns (5) and (6) of Table 5. Only in the highly competitive enterprises listed in column (5), there is a positive correlation between key subordinate governance and accounting conservatism at the significance level of 1%, which further verifies Hypothesis 2.

### Robustness Test

As for the relationship between the governance of key subordinate and accounting conservatism, there may be the problem of omitted variables, that is, impact on both explanatory variables and explained variables. To alleviate this endogeneity problem, the Propensity Score Matching (PSM) method was used to test endogeneity. The property right of the treatment group (state-owned enterprises) and the control group (private enterprises) were used as the grouping basis. According to the method of 1:4 nearest neighbor matching, logit regression was used to calculate the propensity matching score, and the control variables selected in the original principal regression were used as the matching criteria for the PSM analysis. Table 6 shows the results of the two groups according to the matching of control variables, and the SD of the two groups is reduced to <10%, indicating that the difference in enterprise characteristics between the treatment group and the control group is small, basically excluding the influence of other non-observable factors. Table 7 shows the matched sample regression results, the explained variable is accounting conservatism, column (1) is the matched full sample, columns (2) and (3) are the non-overconfident group and overconfident group divided based on (1), and the matched regression results remain stable and consistent with the previous ordinary least squares (OLS) regression results.

**Table 6:** PSM test: features of samples before and after matching.

**Variables**	**Mean**		**%bias**	***t*** **-test**	
	**Treated**	**Control**		***t* value**	***p* value**
size	22.964	22.977	−1.1	−0.53	0.60
lev	0.52222	0.53269	−5.3	−2.82	0.01
TBQ	1.7463	1.7662	−1.7	−0.98	0.33
EPS	0.10006	0.10313	−1.6	−0.98	0.33
PPE	0.22873	0.2144	9.0	4.40	0.00
insthold	5.6725	5.8677	−3.0	−1.63	0.10
balance	0.64378	0.65969	−2.2	−1.46	0.15
indep	0.37165	0.36763	7.4	4.13	0.00
HHI	0.08799	0.0915	−2.9	−1.31	0.19

**Table 7:** PSM test: regression results after matching.

**Variables**	**Dependent variable:UCAC**		
	**Full sample**	**Non-Overconfidence**	**Overconfidence**
CGI_comp	0.022^*^	−0.002	0.047^***^
	(2.06)	(−0.05)	(5.83)
Constant	−0.008	0.019	−0.024
	(−0.22)	(0.63)	(−0.50)
Controls	Yes	Yes	Yes
Year	Yes	Yes	Yes
Ind	Yes	Yes	Yes
N	12,014	2,382	4,643
Adj R^2^	0.177	0.189	0.176

***, **, and * refer to significance at 1%, 5%, and 10%, respectively.

In this article, the two-stage instrumental variable (IV) method is used to estimate the endogeneity of reverse causality. On one hand, the industry median level of key subordinate governance can maintain a certain correlation with the company level of key subordinate governance, and on the other hand, it is exogenous and relatively unrelated to the error term. Therefore, the industry median level of the key subordinate governance (*comp_med pca_med*) was used as the tool variable. Columns (1) and (2) are *comp_med* as a tool variable, and columns (3) and (4) are *pca_med* as a tool variable. The regression results are shown in Table 8. The significance level of key subordinate governance and accounting conservatism is above 10%, which better alleviates the possibility of reverse causality under endogeneity problems. The robustness of the empirical results is further strengthened.

**Table 8:** IV test: regression results.

**Variables**	**Dependent variable: UCAC**			
	**Independent variable:**		**Independent variable:**	
	**CGI_comp**		**CGI_pca**	
	**1st-stage**	**2nd-stage**	**1st-stage**	**2nd-stage**
CGI_comp		0.259^**^		
		(2.57)		
CGI_pca				0.098^*^
				(1.75)
comp_med	0.610^***^			
	(10.80)			
pca_med			0.663^***^	
			(14.38)	
Constant	0.352^***^	−0.196^***^	0.450^***^	−0.110^***^
	(7.34)	(−3.06)	(7.17)	(−2.39)
Controls	Yes	Yes	Yes	Yes
Year	Yes	Yes	Yes	Yes
Ind	Yes	Yes	Yes	Yes
N	17,742	17,742	17,742	17,742
Adj R^2^	0.164	0.151	0.119	0.170

***, **, and * refer to significance at 1%, 5%, and 10%, respectively.

## Discussion

### Research Conclusion

This article studies the relationship between key subordinate governance and accounting conservatism and examines the moderating mechanism based on CEO overconfidence. The empirical results show that key subordinate governance has a positive effect on accounting conservatism. This article also finds that CEO overconfidence can positively moderate the relationship between key subordinate executive governance and corporate accounting conservatism. In the heterogeneity analysis involving the internal property rights and the external competitive environment of enterprises, the overconfidence moderating mechanism in private enterprises and enterprises with high industry competition is more significant, which can better counterbalance CEO overconfidence and improve accounting conservatism.

### Theoretical Contribution

This article has the following research contributions. First, this article improves the measurement of key subordinate executive governance. This article uses the promotion mechanism and practical restraint mechanism. Based on institutional promotion incentive and practical supervision ability, this article optimizes the measurement framework of key subordinate executive governance. Second, this article refines the subject of key subordinate executives. This article emphasizes the special status of VP after CEO. In this article, key subordinate executives are clearly selected as VPs, which improve the accuracy of the governance mechanism of key subordinate executives. Third, this article expands the governance role of key subordinate executives. This article links the prudent attitude of key subordinate governance with the aggressive psychology of CEO overconfidence and discusses the governance path of key subordinate governance to balance CEO overconfidence to ensure accounting conservatism. In addition, considering the governance effect under different property rights and different industry competition environments, the mechanism of key subordinate executive governance on accounting conservatism is better clarified.

### Managerial Implications

Based on this study, attaching importance to the governance status of key subordinate executives is conducive to improving the corporate accounting information quality. This article may provide some managerial implications for the future corporate governance policy reform. First of all, based on the interdependent and mutual restrictive human resources allocation, enterprises should optimize the power balance environment of the TMT, and make the key subordinate executives have more opportunities to display their governance. Second, enterprises should pay attention to the human resources incentives of key subordinate executives, guarantee competitive welfare benefits, create promising promotion mechanism, thus enhancing the governance motivation of key subordinate executives.

### Limitations and Future Research

Although the abovementioned research has enriched the research results of corporate governance and accounting conservatism, there are still some deficiencies. Firstly, the analysis of the governance path for the governance of key subordinates is not clear enough in this article, and the scenario construction of how key subordinate executives exert their own supervision ability and reflect their own supervision power needs further consideration. Furthermore, the measurement of overconfidence needs to be improved, and the key subordinate governance role under other related CEO psychological activities is not explored. At last, the theoretical excavation of relevant disciplines is not thorough enough. In the future, it is necessary to continue to explore fields, such as sociology, psychology, and even Confucianism, so as to better identify and analyze the interaction between key subordinate executives and CEO.

## Data Availability Statement

The original contributions presented in the study are included in the article/supplementary material, further inquiries can be directed to the corresponding authors.

## Author Contributions

FW designed the research method, collected and analyzed the data, and wrote the manuscript. XK was responsible for the conceptualization of the idea and constructed the fundamental theory. Both authors contributed to this article and approved the submitted version.

## Funding

This study was supported by the Bottom-up Internal Governance of TMT: Can the Second Wolf Effectively Supervise the Leader Wolf, which is a Project of the National Natural Science Foundation of China (71962018), and was also supported by the Key Research Base Project of Humanities and Social Sciences in Universities of Jiangxi Province (JD16025).

## Conflict of Interest

The authors declare that the research was conducted in the absence of any commercial or financial relationships that could be construed as a potential conflict of interest.

## Publisher's Note

All claims expressed in this article are solely those of the authors and do not necessarily represent those of their affiliated organizations, or those of the publisher, the editors and the reviewers. Any product that may be evaluated in this article, or claim that may be made by its manufacturer, is not guaranteed or endorsed by the publisher.
